# Optic Neuropathy as a Rare Ocular Complication of Acute Pancreatitis: A Case Report

**DOI:** 10.7759/cureus.54234

**Published:** 2024-02-15

**Authors:** Ramesh Rana, Ashwin Thakali, Umid Kumar Shrestha, Rabin Sharma, Senny Chapagain

**Affiliations:** 1 Department of Gastroenterology and Hepatology, Nepal Mediciti Hospital, Lalitpur, NPL; 2 Department of Ophthalmology, Nepal Mediciti Hospital, Lalitpur, NPL

**Keywords:** case report, complication, purtscher’s retinopathy, optic neuropathy, acute pancreatitis

## Abstract

Acute pancreatitis is a pancreatic inflammatory disorder that often leads to multi-organ dysfunction associated with systemic inflammatory response. Optic neuropathy is an extremely rare ocular manifestation that can occur especially in alcoholic pancreatitis most likely due to ischemic complications and is a vision-threatening condition that has to be recognized early as it can cause permanent vision loss. In this case report, a 51-year-old lady, an occasional consumer of alcohol, post-cholecystectomy status, presented with severe abdominal pain of four days' duration associated with multiple episodes of vomiting. She was diagnosed with moderate to severe acute pancreatitis and needed ionotropic support initially. She had improvement in gastrointestinal symptoms. However, she had left peri-orbital pain and lacrimation with blurring of vision on Day 11 of illness. Subsequently, an ophthalmic evaluation revealed optic disc oedema and a mild decrease in visual acuity but normal visual field and colour vision. Therefore, left optic neuropathy was diagnosed and a high-dose oral steroid was started on a tapering dose. Follow-ups after four and 12 weeks showed significant improvement in optic disc oedema and other symptoms. Therefore, though optic neuropathy is rarely reported in acute pancreatitis, it has to be considered in clinical practice along with Purtscher-like retinopathy, which presents with ocular symptoms in acute pancreatitis.

## Introduction

Acute pancreatitis is a pancreatic inflammatory disorder often associated with a systemic inflammatory response that can lead to multi-organ dysfunction. Ocular complications are rare in acute pancreatitis and are recognized as Purtscher-like retinopathy. Uncommonly, cases of acute pancreatitis present with sudden loss of vision as a result of complement system activation which occurs due to the activated pancreatic enzymes. The activated complement system causes granulocyte aggregation and subsequently retinal vasculopathy due to retinal micro-embolization [1, 2]. This is a vision-threatening condition and needs to be identified early. However, there is no evidence of definitive treatment so far. The prognosis may vary, however, there is a chance of spontaneous resolution in some cases [3]. Optic nerve involvement in acute pancreatitis is even rarer.

We present a case of unilateral optic neuropathy in acute necrotizing pancreatitis which, to our knowledge, has only once been reported before by Chylova et al. [4].

## Case presentation

We report a 51-year-old lady who presented in our hospital after four days of abdominal pain which was acute onset, generalized, radiating to the back, and not relieved by taking analgesics. The pain was associated with non-bilious and non-blood mixed multiple episodes of vomiting. She had a history of cholecystectomy for symptomatic cholelithiasis five years back. She was an occasional social drinker, however, there was no history of binge drinking or alcohol intake >14 units/ week or alcohol intake within two weeks of the abdominal pain. Besides, she didn't have a significant medical or surgical history of systemic diseases. 

On examination, her pulse rate was 96 beats per minute, blood pressure of 90/60 mmHg (on inotropic support which was eventually withdrawn after fluid challenge), oxygen saturation of 92% at room air and respiratory rate of 22 breaths per minute. She was non-icteric and without pallor. On abdominal examination, three healed surgical scars were seen, the abdomen had mild tenderness over the epigastrium and umbilical region with negative for rebound tenderness or Murphy’s sign, no organomegaly, and bowel sound was normal. The respiratory and cardiac examinations were normal.

Her laboratory findings showed mildly elevated total bilirubin (1.8mg/dl) and indirect bilirubin 1.5mg/dl with normal transaminase (alanine aminotransferase of 23U/L and aspartate aminotransferase of 26U/L) and alkaline phosphatase levels (84U/L), gamma-glutamyl transferase (GGT) of 30U/L, and reduced albumin 2.5g/dl. Amylase was 392U/L, lipase was 1063U/L, calcium was 7.10mg/dl, and C-reactive protein of 254.7mg/L. There was a normal renal function test (urea: 11mg/dl, creatinine: 0.7mg/dl, sodium: 137mmol/L and potassium: 4.0mmol/L), lipid profile (triglycerides: 65mg/dl, total cholesterol: 112mg/dl, high-density lipoprotein: 31mg/dl, low-density lipoprotein: 64.92, and very low-density lipoprotein: 13mg/dl), and blood sugar level (81mg/dl).

**Figure 1:**
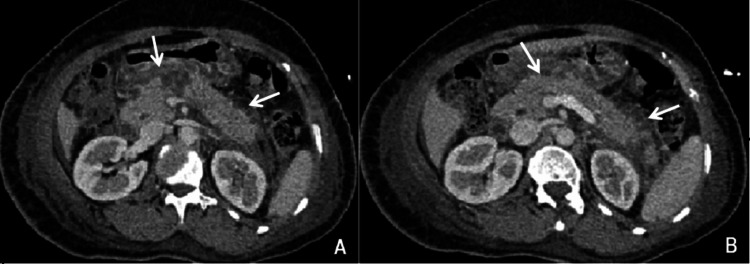
A and B showed mildly and diffusely swollen pancreas with a non-enhancing hypodense area in the caudal aspect of the proximal part of the body of the pancreas with minimal peripancreatic fluid collection and features suggesting acute necrotizing pancreatitis.

**Figure 2:**
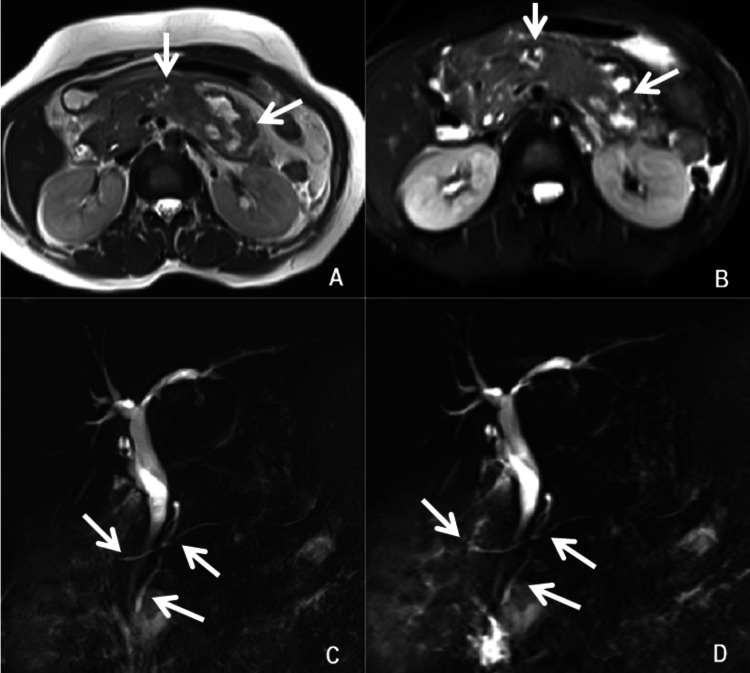
A and B showed bulky distal parts of the body and tail of the pancreas with small collections in the peripancreatic area. C and D showed variant pancreatic ductal anatomy with drainage of the pancreatic duct in both major and minor papillae.

Liver ultrasonographic findings of the prominent common bile duct and prominent pancreatic duct were suggestive of acute pancreatitis. She needed ionotropic support initially despite adequate fluid challenge and was managed conservatively in the intensive care unit (ICU). Contrast-enhanced computed tomography (CECT) abdomen was performed on Day 5 of admission as the patient was feverish and needed oxygen support of 3-5 litres/min. It revealed an enlarged liver of 18.4cm in size and a mildly dilated proximal common bile duct of 11.5mm with a smooth distal tapering without any overt cause. The pancreas was mildly and diffusely swollen with approximately 22.6 x 19.8mm size non-enhancing hypo-dense area noted in the caudal aspect of the proximal part of the body of the pancreas. Soft tissue stranding in the peripancreatic fat, minimal peripancreatic fluid collection, and minimal fluid collection were also noted in sub-hepatic space, left para-colic gutter and pelvis, the pancreatic duct was not dilated. The features were suggestive of acute necrotizing pancreatitis with the minimum peri-pancreatic fluid collection (Figure 1) and minimum fluid collection in the left sub-hepatic space left para-colic gutter and pelvis (modified CT severity index: 8/10); bilateral moderate pleural effusion with lower lobe atelectasis. She was transferred to the ward on Day 9 of illness. Further, magnetic resonance cholangiography (MRCP) was consistent with the evidence of necrotizing pancreatitis with few small collections in the peripancreatic area with bulky pancreatic tail and distal part of pancreatic body, slightly prominent cystic duct stump with post cholecystectomy status. In addition, variant pancreatic ductal anatomy with drainage of the pancreatic duct in both major and minor papillae was seen suggestive of pancreatic division (Figure 2). 

**Figure 3:**
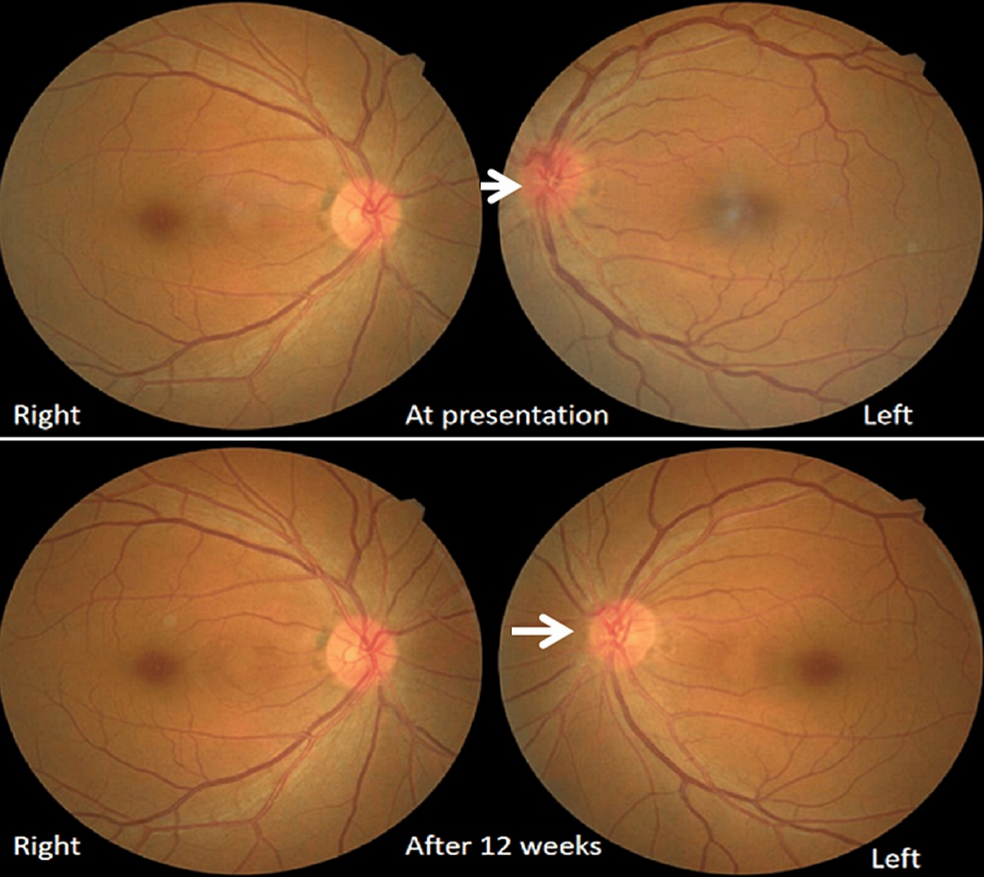
Fundoscopic examination showed optic disc oedema with mildly tortuous retinal vessels in the left eye and normal on the right at presentation and normal on both eyes after 12 weeks.

On Day 11 of illness, she complained of left-sided blurry vision, lacrimation, and mild retro-orbital pain. Ocular examination revealed optic disc oedema with mildly tortuous retinal vessels in the left eye (Figure 3). The best corrected visual acuity was 6/6p in the right eye and 6/9 in the left. Measurement of pupil size showed anisocoria, 3mm in the right eye and 5mm in the left eye under equal illumination conditions. Intraocular pressure (IOP) was raised in both eyes, 22 mmHg and 32 mmHg in the right and left eye respectively. However, the visual field and colour vision were normal. Therefore, a secondary complication of acute necrotizing pancreatitis, optic neuropathy was diagnosed. Giant cell arteritis was ruled out as she had no symptoms like jaw claudication, scalp or temporal tenderness or polymyalgia and erythrocyte sedimentation rate was normal. Additionally, alcohol-induced optic neuropathy was ruled out as GGT was normal with no recent alcohol intake history. 

The oral steroid was started on a tapering dose for one month. Eye drops of timolol on the right eye and a combination of timolol and brimonidine eye drops on the left eye were also added to control intraocular pressure (IOP). On the following day, IOP was well controlled at 16 mm Hg in the right eye and 14 mm Hg in the left eye. Thus, timolol was continued only in the left eye for one week. On one week follow-up, the patient had a remarkable improvement in the symptoms without any pain. On eye examination, best-corrected visual acuity (BCVA) was 6/6p in both eyes, with reduced optic disc swelling, and no anisocoria. The IOP was 15 mmHg and 13 mmHg in the right and left eye, respectively.

On four weeks follow-up, she was asymptomatic. Bilateral BCVA was 6/6p, no anisocoria on pupillary examination, and IOP of 15mmHg and 14mmHg in the right and left eyes, respectively. Endoscopic ultrasound revealed no pancreaticobiliary abnormalities. Additionally, serum IgG4 was normal.

## Discussion

Optic neuropathy or Purtscher-like retinopathy, a rare ocular complication, is characterized by sudden visual loss with multiple areas of retinal whitening in the posterior pole of the eye and is a non-traumatic entity associated with complement-activating systemic diseases such as acute pancreatitis, connective tissue disorders, renal failure, childbirth, bone marrow transplantation, fat embolism syndrome, and the Valsalva manoeuvre [3, 4]. In pancreatitis, it is presumably caused by proteases released during inflammation or injury that provoke fat micro embolisation of retinal and choroidal arterioles after severe trauma characterized by retinal ischaemia and haemorrhage. The common fundoscopic findings are cotton-wool spots and intra-retinal haemorrhage. Purtscher flecken are pathognomonic; however, these are seen in only 50% of cases [5]. Flecken occurs due to occlusion of the precapillary arterioles and is characterized by intraretinal whitening. Similar pathology involving the optic nerve can lead to ischemic optic neuropathy, however, this is extremely rare, with only one case report being reported in the literature [4].

Generally, the patient with Purtscher-like retinopathy or optic neuropathy presents with the sudden onset of visual impairment or blindness, bilateral in up to 60% of cases [5]. However, in our case, our patient had minimal unilateral visual impairment with peri-orbital pain. Purtscher-like retinopathy or optic neuropathy is more prevalent in alcoholic than nonalcoholic pancreatitis; the reason behind this is unknown. Hypercoagulable states have been associated with nonarteritic anterior ischemic optic neuropathy [6]. The onset of ocular symptoms may vary from the days to months from the day of pancreatitis. The poor prognostic factors for visual improvement are choroidal hypoperfusion, optic disc swelling or massive Purtscher flecken [5]. The definite treatment protocol for Purtscher retinopathy has not been established so far, and several studies on corticosteroid treatment did not demonstrate statistically significant improvements [3, 5]. However, a high-dose steroid or prednisolone 1.5mg/kg/day dose improved symptoms [7, 8], and in our case as well, she had significant improvement in symptoms with 1mg/kg/day dose. In contrast, a case is reported in the literature, where optic neuropathy treated with a high dose of steroid (methylprednisolone) caused drug-induced acute pancreatitis [4, 9].

The strength of our case report is that we were able to diagnose optic neuropathy early even though she had milder ocular symptoms. The early ophthalmic evaluation helped to reach the diagnosis of left-sided optic neuropathy despite the rare presentation in acute pancreatitis. Moreover, early high-dose prednisolone was started with timolol and brimonidine eye drops and close monitoring of the case to observe the outcome properly.

There were some limitations though we were able to diagnose and manage it early. We could not ascertain the cause of pancreatitis, however, it could be associated with pancreatic division as per the MRCP findings. Further, IgG-4 and EUS were negative for autoimmune and biliary causes, respectively. We didn’t perform the magnetic resonance imaging of the brain due to the patient's financial limitations, however, in our opinion, it did not contribute a great deal to the management of optic neuritis. Also, we did not check the vitamin levels. 

## Conclusions

Optic neuropathy is a rare ocular complication that can occur in acute pancreatitis; however, we need to exclude other causes of raised IOP, optic neuropathy, or acute pancreatitis such as alcohol, hypovitaminosis, connective tissue disorders or ischemic causes. Though this condition has a spontaneous resolution, it should not be missed or neglected as it can cause permanent ocular damage. 
